# Editorial: Health and safety issues of employees in family firms

**DOI:** 10.3389/fpubh.2023.1102736

**Published:** 2023-02-02

**Authors:** Muhammad Waseem Bari, T. Ramayah, Francesca Di Virgilio, Emilia Alaverdov

**Affiliations:** ^1^Lyallpur Business School, Government College University Faisalabad, Faisalabad, Pakistan; ^2^School of Management, Universiti Sains Malaysia (USM), George Town, Malaysia; ^3^Department of Information Technology and Management, Daffodil International University, Dhaka, Bangladesh; ^4^Fakulti Ekonomi dan Pengurusan (FEP), Universiti Kebangsaan Malaysia (UKM), Bangi, Malaysia; ^5^Azman Hashim International Business School, Universiti Teknologi Malaysia (UTM), Johor Bahru, Malaysia; ^6^Applied Science Private University (ASU), Amman, Jordan; ^7^University Center for Research and Development (UCRD), Chandigarh University (CU), Sahibzada Ajit Singh Nagar, Punjab, India; ^8^Department of Economics, University of Molise, Campobasso, Italy; ^9^Faculty of Law and International Relations, Georgian Technical University, Tbilisi, Georgia

**Keywords:** health, safety, family firms, workplace, small- and medium-size enterprises (SMES)

Worker health and safety issues have been the subject of extensive study, including when, why, and how they arise (1, 2). However, the reasons behind the health and safety issues of the employees working in family-owned firms are still under-explored. A business or entity owned and managed by the members of a single-family is known as a “family business firm” (3). The family firms' operations and ownership are transferred from generation to generation. Usually, more than one generation is involved in the day-to-day operations and management of the business. The family firms perform with limited financial and other resources and have a small network (4). Thus, family firms have limited resources to facilitate their employees at the workplace, such as improper lighting, hygiene issues, an overfilled workplace, and the absence of facilities such as safety tools and safety instruction boards. Healthy food and clean water are also serious issues for workers at the workplace in family firms (5).

In underdeveloped and developing countries, the ratio of employees working in family firms and SMEs is very high compared to the corporate sector (6). Several studies have provided evidence of the relationships between working conditions, employees' health and safety, and productivity (7, 8). Therefore, it is vital to develop a system to address the health and safety issues at the workplace in family firms. Researchers describe the consequences of the issues regarding the health and safety of the employees, such as low performance of workers and the firm, a high rate of absenteeism, a poor rate of productivity, a high turnover rate of employees, stress, anxiety, disengagement, and psychological issues (9). In family firms, the limited focus of the managers, lack of financial resources, and absence of the workers' leadership play a significant role in the development of health and safety issues in employees. In this Research Topic, a total of 20 manuscripts were submitted; eight manuscripts were rejected due to quality issues, and 12 articles were accepted. The following paragraphs explain the themes and contributions of the articles that are published in the Research Topic with the title “Health and Safety Issues of the Family Firms' Workers.”

*First*, the article explains the employees' performance during COVID-19 in small family firms. Post-COVID-19, these small firms tried to regain their market share, but they were not significantly successful, and a financial crisis rose in these family firms (10). Even so, these organizations failed to provide financial benefits to their workers. According to the authors, in these types of scenarios, social and psychological rewards can aid management. Therefore, the article investigated how top management can enhance employees' performance by using social and psychological rewards in the absence of financial rewards. By using a stratified sampling technique, the data were collected from 250 employees working in small family firms. The findings confirm that psychological rewards help increase employees' performance. However, social rewards do not have a significant impact on employees' performance.

*Second*, drawing on the appraisal theory of emotions, the paper investigates the impact of ethical leadership behaviors on employees' negative emotions, i.e., workplace embitterment and the moderating role of core self-evaluation. Data were collected from 398 employees working in Pakistan's public sector universities using a random sampling technique. *Findings* indicate that leaders' ethical behaviors negatively impact employees' workplace embitterment, and workplace embitterment mediates this impact on employees' wellbeing (11). Moreover, employees' core self-evaluation moderates the relationship between leaders' ethical behaviors through workplace embitterment.

*Third*, the COVID-19 pandemic brought several changes to every field of life. The educational system is also affected by this pandemic. The educational institutions shifted their activities from a physical to an online mode. The online educational mode brought about several attitude and behavioral changes in students. Drawing on stress theory, the article investigates the impact of pandemic fear on student performance and anxiety in students as a mediator. Moreover, the article also explored the role of mindfulness as a moderator. The data was collected from HSK teachers working in China. The results confirm that fear of the pandemic negatively impacts students' performance and develops anxiety in students. However, mindfulness has failed to perform its moderating role in the article model. The article also offers several managerial implications and theoretical contributions. *Fourth*, the dynamics of family-owned firms are different from other business entities. As a result, the antecedents and outcomes of family-owned businesses may differ. The purpose of this study is to look into the effect of psychological distance between a non-family member employee and a family member on occupational mental health and psychological safety as a mediator in the relationship. In addition, a proactive personality as a potential moderator is applied in the article. Two approaches, PSL-SEM, and fsQCA are used for data analysis. The results confirm the partial mediating role of psychological safety.

*Fifth*, the global health emergency (COVID-19 pandemic) raised serious issues regarding health and safety issues for the employees of family-owned firms. The paper explains how Chinese family enterprises face problems regarding employees' performance, health, and safety after dynamic changes at the workplace due to COVID-19. Drawing on game theory, the article researched Chinese family-owned firms. The data were collected from the firms in the list provided by a 3rd party CSR rating agency (SynTao Green Finance). According to the article's findings, family businesses are less likely than non-family businesses to fulfill workers' health and safety responsibilities. From the operational perspective, family business firms are gradually improving facilities for their workers; this process, however, is “U” shaped. The results indicate that more effective policies and stakeholder monitoring are required to solve the health and safety issues of the employees. It is also important to provide awareness to the workers regarding their legal and professional rights.

*Sixth*, the paper examines the impact of testing fear and less social connectedness on employees' health. In addition, the scholars used psychological strain as a mediator between less connectedness, testing fear, and employees' health and performance. Data for the empirical investigation were collected from employees working in China's electronic industry using a convenience sampling approach. The partial least squares structural equation modeling approach (PLS-SEM) was used for data analysis with the Smartpls-3 software. The results of the article indicate that COVID-19 testing fear impacts the employees' health negatively, but social connectedness does not significantly impact employees' health. However, psychological strain significantly mediates the relationship between testing fear, less social connectedness, and employees' health. The paper also provided valuable insights for organizational management to develop a healthy and positive working environment and adopt healthy behaviors among their workers, which ultimately fosters their job performance.

*Seventh*, human resources play a strategic role in making or breaking a brand. The COVID-19 pandemic has made organizations and their management realize the importance of health safety. Health-oriented strategies play a significant role in boosting employees' trust and wellbeing. The paper investigates the role of organizational health-oriented strategies in improving employees' job performance using social exchange theory. The article also used employees' psychological wellbeing and trust as mediators in the above-said relationship. In addition, the article also investigated the moderating role of perceived medical mistrust. By using a random sampling technique, the authors collected the data from the employees working in the textile sector of China. The article proved that health-oriented strategies positively increase employees' trust and psychological wellbeing, leading to improved employee performance. The article also found a significant role in medical mistrust between psychological wellbeing and employees' performance but failed to moderate the relationship between trust and job performance. Moreover, the findings of the present article also serve the literature by providing important theoretical and practical implications.

*Eighth*, the paper evaluates the impact of employee loneliness, psychological distress, and job uncertainty on employee-based brand equity. In addition, emotional exhaustion has been used as a mediator in the above-said relationship. For empirical evidence, the data were collected from 459 employees working in clothing brands in China. Results, job uncertainty, and psychological distress negatively impact employees based on brand equity. However, employee loneliness has no impact on employees based brand equity. *Nine*, the article was conducted on the employees of 25 readymade garment factories in Dhaka, Narayanganj, and Gazipur industrial areas of Bangladesh on a random sampling basis. The impact of occupational stress on employees' health risks was measured. As the result, occupational stress has an impact on employees' health. However, female workers were more affected than male workers.

*Tenth*, the paper clarifies the types of hazards that exist in dental hospitals, as well as worker stress. The findings described the occurrence of ergonomic, physical, biological, and chemical hazards in the workplace. Ergonomic hazards have the highest occurrence, and chemical hazards have the least occurrence. *Eleventh*, the article is about *the janitorial staff*. The paper aims to measure the impact of occupational safety practices to prevent COVID-19 transmission and associated factors on the janitorial staff of an Ethiopian university. The results explained that occupational safety practices regarding COVID-19 were in practice among 53.9% of the janitorial staff. *Twelve*, based on university faculty members, the paper investigates the impact of the workplace environment on employee performance, with a particular emphasis on the role of employee achievement striving ability as a mediator. The results confirm the hypotheses of the article.

Overall, this Research Topic is very helpful in understanding the antecedents and consequences of employees' health and safety issues and in providing suitable solutions. The topics in this Research Topic are constructive for human resource management students, research scholars, human resource management faculty, and professional consultants in human resource management, health, and safety.

## Author contributions

All authors listed have made a substantial, direct, and intellectual contribution to the work and approved it for publication.

## References

[1] Laberge M Ledoux E. Occupational health and safety issues affecting young workers: a literature review. Work. (2011) 39:215–32. 10.3233/WOR-2011-1170 21709358

[2] Moyce SC Schenker M. Migrant workers and their occupational health and safety. Annu Rev Public Health. (2018) 39:351–65. 10.1146/annurev-publhealth-040617-013714 29400993

[3] Lude M Prügl R. Why the family business brand matters: brand authenticity and the family firm trust inference. J Bus Res. (2018) 89:121–34. 10.1016/j.jbusres.2018.03.040

[4] Van den Heuvel J Van Gils A Voordeckers W. Board roles in small and medium-sized family businesses: performance and importance. Corp Gov Int Rev. (2006) 14, 467–485. 10.1111/j.1467-8683.2006.00519.x

[5] Campopiano G De Massis A Cassia L. Corporate social responsibility in family versus non-family enterprises: an exploratory article. In:Lundström A Zhou C von Friedrichs Y Sundin E, editors. Social Entrepreneurship. New York, NY: Springer (2014), p. 113–54. 10.1007/978-3-319-01396-1_6

[6] Abor J Quartey P. Issues in SME development in Ghana and South Africa. Int Res J Finance Econ. (2010) 39:215–28.

[7] Fernández-Muñiz B Montes-Peón JM Vázquez-Ordás CJ. Relation between occupational safety management and firm performance. Sa Sci. (2009) 47:980–91. 10.1016/j.ssci.2008.10.022

[8] Lundstrom T Pugliese G Bartley J Cox J Guither C. Organizational and environmental factors that affect worker health and safety and patient outcomes. Am J Infect Control. (2002) 30:93–106. 10.1067/mic.2002.119820 11944001

[9] Ongori H Agolla JE. Occupational stress in organizations and its effects on organizational performance. J Manag Res. (2008) 8:123–35.

[10] Le Breton-Miller I Miller D. Family businesses under COVID-19: inspiring models–sometimes. J Fam Bus Strategy. (2022) 13:100452. 10.1016/j.jfbs.2021.100452

[11] Michailidis E Cropley M. Exploring predictors and consequences of embitterment in the workplace. Ergonomics. (2017) 60:1197–206. 10.1080/00140139.2016.1255783 27801614

